# Performance Evaluation of Foamed Concrete with Lightweight Aggregate: Strength, Shrinkage, and Thermal Conductivity

**DOI:** 10.3390/ma17153869

**Published:** 2024-08-05

**Authors:** Jiaying Wei, Tianyu Wang, Ying Zhong, Yi Zhang, Christopher K. Y. Leung

**Affiliations:** 1 Department of Civil and Environmental Engineering, Hong Kong University of Science and Technology, Clear Water Bay, Hong Kong, China; jwei@connect.ust.hk; 2 China State Construction Engineering (Hong Kong) Limited, Hong Kong, China; jacky_zhong@cohl.com (Y.Z.); zy_hk@cohl.com (Y.Z.); 3 China State Construction International Medical Industry Development Co., Ltd., Hong Kong, China

**Keywords:** foamed concrete, lightweight aggregate, drying shrinkage, thermal insulation, building energy simulation

## Abstract

Lightweight concrete offers numerous advantages for modular construction, including easier construction planning and logistics, and the ability to offset additional dead loads induced by double-wall and double-slab features. In a previous study, authors proposed incorporating lightweight aggregate into foamed concrete instead of adding extra foam to achieve lower density, resulting in lightweight concrete with an excellent strength-to-density ratio. This paper further investigated the performance aspects of foamed concrete with lightweight aggregate beyond mechanical strength. To evaluate the effect of aggregate type and foam content, three mix compositions were designed for the lightweight concrete. Specimens were prepared for experimental tests on thermal conductivity and drying shrinkage of lightweight concrete. Results showed that while both the increase in foam volume and the incorporation of lightweight aggregate led to higher drying shrinkage, they also contributed to improved insulating properties and reduced potential of cracking. Using typical multi-storey modular residential buildings in Hong Kong and three other Chinese cities as case studies, simulations were performed to assess potential savings in annual cooling and heating loads by employing the proposed lightweight concrete. These findings demonstrate the practical benefits of using foamed concrete with lightweight aggregate in modular construction and provide valuable insights for further optimization and implementation.

## 1. Introduction

In recent years, modular construction, a process in which fully furnished units are assembled in a factory before installation on-site, has gained significant attention in regions such as Hong Kong. This attention is due to the method’s potential to enhance construction efficiency, safety, and quality, while reducing environmental impacts.

Buildings account for approximately a third of global energy consumption and carbon emissions [1,2]. These emissions are categorized into embodied carbon (from material production, transportation and construction) and operational carbon (from energy use during a building’s lifespan). Previous research has demonstrated that prefabrication can reduce both embodied [3,4,5,6] and operational carbon emissions [6,7,8], with further reductions achievable through the selection of materials with high insulating properties [6].

Lightweight concrete has been introduced into this context to reduce the self-weight of reinforced concrete structures, an essential property for modular construction. This is particularly important when double-wall and double-slab features add extra weight and pose challenges to site conditions and load-bearing structures. Moreover, it offers enhanced transportation and logistics management due to its lighter modules. Lightweight concrete may also contribute to a better sustainability by reducing the depletion of natural aggregates [9].

Lightweight concrete achieves reduced density through the introduction of additional voids in the cementitious matrix (foamed concrete) or through the use of highly porous aggregate (lightweight aggregate concrete). Strength correlates closely with density [10,11]. Studies have pursued higher strength at lower density by optimizing microstructure (e.g., smaller, uniform pores) in foamed concrete [10,12,13,14,15,16,17,18,19,20,21,22]. Recently, Wei et al. combined foamed concrete with lightweight aggregate to lower weight while maintaining strength through pore structure control, avoiding excessive foaming [23].

The results in Ref. [23] demonstrated mechanical feasibility for modular buildings. However, the insulating properties relevant to operational carbon emissions require further study. Thermal conductivity of concrete determines the rate of heat loss through roofs and walls that make up the envelope of a building, which in turn affects the building’s energy consumption. To reduce the high consumption of cooling and heating energy for households, a common practice is to adopt structural concrete with lower thermal conductivity. Since thermal conductivity decreases with increasing porosity, it is well established that the thermal conductivity of concrete is positively associated with its unit weight [24,25]. Therefore, compared to conventional concrete, both foamed concrete and lightweight aggregate concrete exhibit much lower conductive properties and are frequently used in the building envelopes for thermal insulation purposes, improving the energy efficiency of buildings [11,25,26,27,28,29,30,31,32]. On that account, the lightweight concrete developed in Ref. [23] which also exhibits sufficient strength, has the potential to be a promising structural material for the construction of passive, energy-saving modular buildings.

Dimensional stability must also be characterized given its implications for durability. Shrinkage-induced cracking allows the penetration of water and the ingression of aggressive substances like chlorides and carbon dioxide. These substances can accelerate the corrosion of steel reinforcement and shorten the service life of buildings. Drying shrinkage occurs when moisture content decreases towards the member surface. Different drying shrinkage mechanisms are triggered according to the level of pore relative humidity, including capillary pressure, disjoining pressure, surface tension, pore blocking and movement of interlayer water [33]. It is crucial to monitor the drying shrinkage for foamed concrete because of its high porosity and the large specific surface area of pores [11,34]. The addition of aggregate is a common means to resist the contraction of cement paste. However, studies have concluded that the drying shrinkage of lightweight aggregate concrete is usually higher compared to conventional concrete because lightweight aggregate is much less rigid due to its highly porous nature [35,36,37,38,39]. In the existing literature, the drying shrinkage of lightweight aggregate concrete and foamed concrete has been reported to range between 600 and 1200 με, and 600 and 3000 με, respectively [34,40,41,42].

This study centers on a specific type of lightweight concrete, developed in earlier research to provide adequate strength while reducing weight, a crucial factor in modular construction [23]. This paper aims to further justify and endorse its use by investigating properties beyond mechanical strength: thermal conductivity and drying shrinkage. Experimental tests are conducted in these regards, considering the influence of aggregate types and foam content. Furthermore, simulations are performed to approximate potential reductions in annual cooling and heating loads in a typical multi-storey modular building when the proposed lightweight concrete replaces conventional concrete. Our study aims to deliver a more holistic understanding of the benefits of lightweight concrete with aggregate and contribute to its further improvement for real-world applications.

## 2. Materials and Methods

### 2.1. Materials

The following constituent materials were used in this study to produce lightweight concrete: (1) Portland cement, CEM I-52.5N (3.1 S.G.) conforming to BS EN 197-1:2011, (2) Fly ash (2.4. S.G.), from a local power plant, (3) Silica fume (2.3 S.G.), from Elkem Materials, in the form of uncompacted micro silica powder, (4) Silica sand (2.6 S.G.), sieved to remove particles larger than 0.85 mm, (5) Lightweight aggregate (1.65 S.G. in saturated surface dry condition), manufactured from recycled industrial by-products including fly ash, coal gangue, metallurgical slag and tailing sand, by a factory in the mainland China. The lightweight aggregate was sieved to remove particles larger than 0.85 mm, (6) Clean tap water, (7) Polycarboxylate-based superplasticizer (ADVA 189), (8) Foam (36 kg/m^3^) was produced by blending the foaming agent (BASF-MasterCell 10) with water and compressed air by predetermined proportions (40 g water to 1 g foaming agent) in a foam generator.

The chemical composition of cement, fly ash, silica fume and lightweight aggregate used in this study are determined using X-ray fluorescence (XRF, Element analyzer, JEOL JSX-3201Z) analysis on the raw material powders and listed in Table 1.

### 2.2. Mix Proportions

The proportions of constituent materials in this paper were designed based on the authors’ previous study [23]. The parameters considered in this study are aggregate type and foam volume. Three mix compositions were prepared to examine the effect of aggregate type (lightweight aggregate, silica sand, and no aggregate) (Table 2). In addition to the materials listed in Table 2, superplasticizer was added to the mix to achieve suitable workability, with the amount adjusted according to visual inspection during mixing. For each mix composition, various contents of foam were added to make concrete of three to four nominal plastic densities, ranging from 1300 kg/m^3^ to approximately 2200 kg/m^3^ (the exact value depends on the density of concrete mix without foam).

### 2.3. Specimen Preparation

Lightweight concrete was produced using the pre-foaming method. To prepare the base mortar, dry materials including cement, silica fume, fly ash and aggregate were mixed for one minute in the mortar mixer before adding water and superplasticizer. Mixing continued for another five minutes until the individual ingredients were fully transformed into a consistent mortar. In the meantime, the foaming agent was mixed with clean tap water by mass ratio of 1:40 and blended with compressed air in the foam generator. Once the generated foam became stable, it was weighed and placed in a portable mixer, where the base mortar was added shortly after. The materials were mixed until the mixture became uniform, with no visible bubbles on the surface. The mixture was then poured into molds of different sizes and covered with plastic sheet for 24 h before demolding. All cubic specimens were weighed to obtain fresh density. Afterwards, samples were placed in the curing chamber at temperature of 23 ± 2 °C and relative humidity of 95 ± 5% till the age of 28 days.

### 2.4. Test Procedure

#### 2.4.1. Compressive Strength and Flexural Strength

For each batch of concrete, three cylinder specimens with size of ϕ50 × 100 mm, three cubic specimens with side lengths of 40 mm and three prism specimens with size of 40 × 40 × 160 mm were prepared for testing at the age of 28 days. The static modulus of elasticity and compressive/flexural tests were conducted in accordance with ASTM C469/C469M-14 [43] and BS EN 196-1 [44] using Autocon 2000 Material Tester and MTS 810, respectively. In view of the small particle size (no more than 0.85 mm for dry materials, due to the absence of coarse aggregate) and pore size (mainly in the range of 10 μm to 1 mm [45]) in the mix composition, smaller-sized specimens were adopted. Foamed concrete specimens of similar size has been commonly used for compressive tests in other recent studies [20,22,23,46,47,48].

#### 2.4.2. Thermal Conductivity

Two plate specimens measuring 150 × 150 × 50 mm were prepared for thermal conductivity measurement of each test group. To simulate the ambient environment of concrete at indoor working conditions, the specimens were placed in a room with a temperature of 25 ± 0.5 °C and humidity of 50 ± 1% humidity after 28 days of moist curing and were tested at a mature age of no less than 90 days. While these conditions do not fully simulate real-world scenarios where one side of a wall is exposed to indoor conditions and the other to external environments, they provide a standardized testing environment.

**Figure 1:**
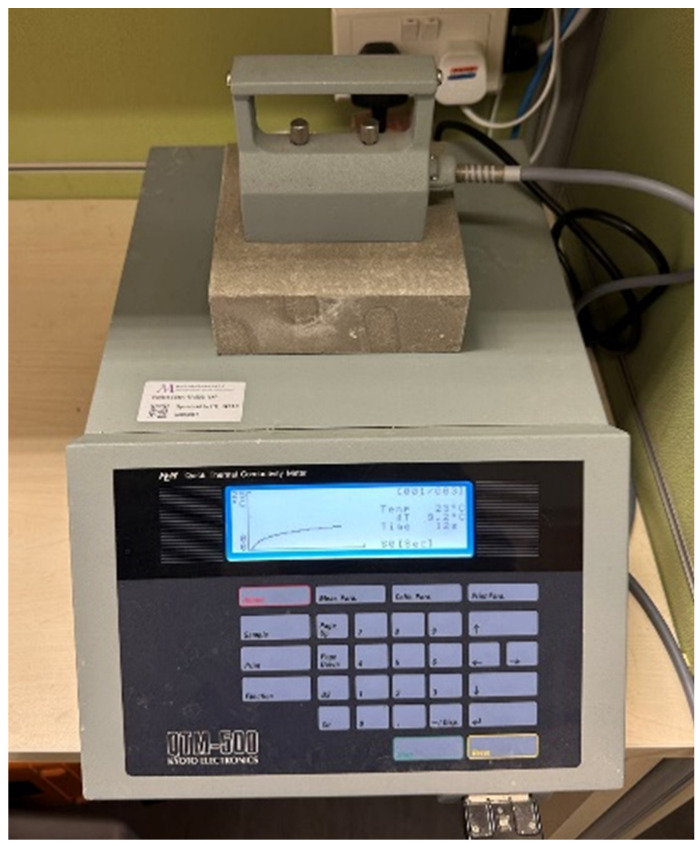
Thermal conductivity test of lightweight concrete using QTM-500.

In this study, the thermal conductivity of lightweight concrete was measured using a QTM-500 quick thermal conductivity meter (Figure 1), which employs the transient hot wire method. A hot wire acts as a linear heat source on the surface of the test material, and the elevated temperature at a specific distance from the wire is monitored [25]. The change of temperature with time (i.e., the transient) is related to the thermal conductivity. The transient method is commonly used for measuring the thermal conductivity of heterogeneous material with moisture content, such as concrete [49]. In addition, the transient method is less time-consuming and requires less expensive and more readily available equipment, making it a more popular option than the steady state method [25,50]. A total of 24 measurements were conducted at different faces of each specimen to obtain more accurate results.

#### 2.4.3. Drying Shrinkage

**Figure 2:**
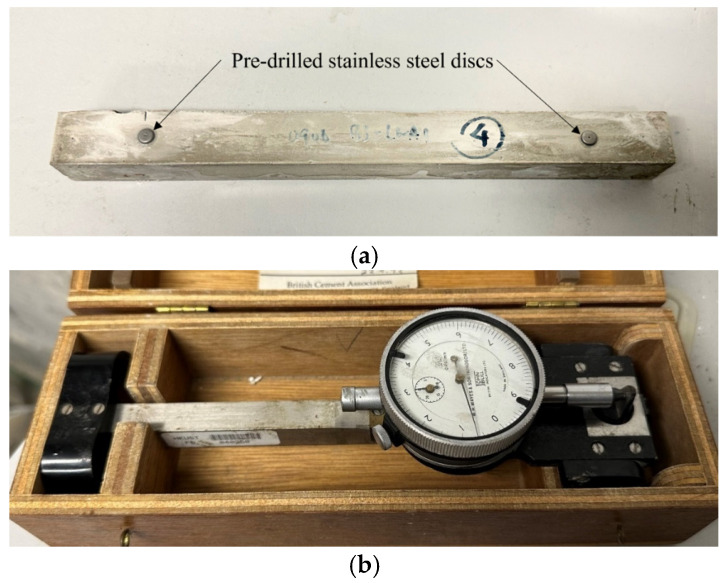
(**a**) prism specimen with Demec points for drying shrinkage measurement; (**b**) Demec gauge.

Three prism specimens measuring 25 × 25 × 285 mm were prepared for drying shrinkage measurement for each test group. After 28 days of curing, pre-drilled stainless-steel discs were attached to the front and back surfaces of each specimen using adhesive. Two discs were placed on each surface, maintaining an initial distance of 200 mm between them (Figure 2a). The day of disc installation was considered day zero. The samples were placed in a room regulated at 25 ± 0.5 °C and 50 ± 1% humidity. The length change between the discs was measured using a Demec gauge every day for the first two weeks, and then at the age of 28 days, 56 days, 84 days, and 90 days (Figure 2b).

## 3. Results

### 3.1. Compressive Strength and Flexural Strength

**Figure 3:**
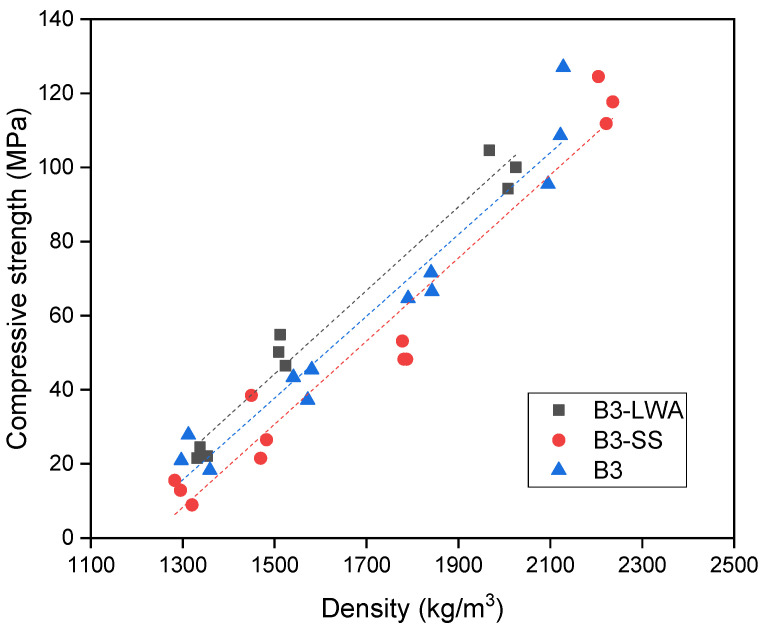
Compressive strength vs. density plot.

**Figure 4:**
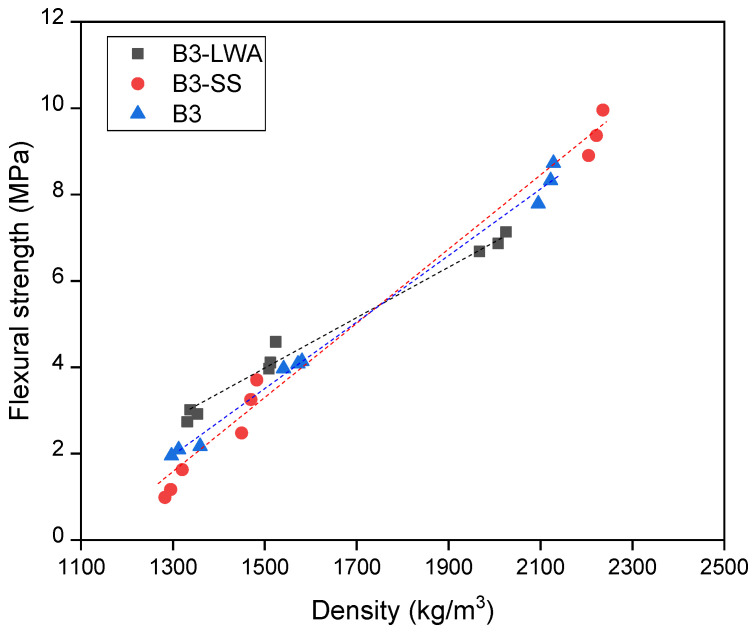
Flexural strength vs. density plot.

The compressive strength of each concrete batch was obtained and plotted against concrete density in Figure 3. Within the studied range of approximately 1300 to 2200 kg/m^3^, the compressive strength exhibits an almost linear relationship with density. This observation concurs with the early work of Hoff (1972), who stated that this relationship should be curvilinear overall, but could be linear within a certain density range [51]. As evident from Figure 3, the trendlines for the three mixes indicate that at equivalent densities, the order of compressive strength is B3-LWA > B3 > B3-SS. This ordering suggests that mix B3-LWA achieves the highest compressive strength-to-density ratio among the three mixes, making it the most efficient in terms of strength per unit weight. A similar trend is observed in the flexural behavior of the mixes. As shown in Figure 4, the flexural strength of all the specimens increases with density. In the non-foamed condition, the order of flexural strength follows the density trend: B3-SS > B3 > B3-LWA. However, when foam is added to reduce the density to 1500 kg/m^3^ and 1300 kg/m^3^, the order of flexural strength changes to B3-LWA > B3 > B3-SS. The B3-LWA group exhibits a slower decrease in flexural strength as density reduces.

The differences between mixes can be attributed to the varying pore structure. To achieve the same density, the required volume of foam for B3-LWA is much lower, because B3-LWA comprises both foam voids and pores within lightweight aggregate. The use of highly porous lightweight aggregate results in smaller pores in matrix that are more uniformly distributed and exhibit significantly lower pore connectivity. On the contrary, B3 and B3-SS are primarily composed of voids that are formed by foam addition, which tend to create large-sized pores acting as ‘weak links’ that are detrimental to the mechanical strength [23]. At similar strength, the matrix density of B3-SS is higher due to the incorporation of silica sand (S.G. = 2.6), and therefore requires more foam than B3.

### 3.2. Thermal Conductivity

**Figure 5:**
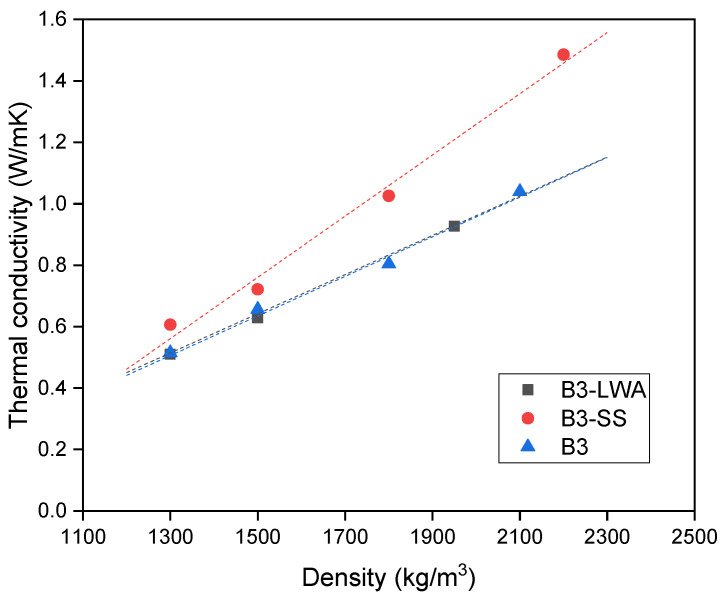
Thermal conductivity vs. density plot.

Figure 5 illustrates the relationship between thermal conductivity and density of lightweight concrete. As expected, a decrease in concrete density leads to a reduction in thermal conductivity, which is demonstrated by a well-fitted linear regression. This trend is attributed to the fact that density is an indicator of porosity, where greater porosity reduces conductivity due to the good insulating properties of the air within pores. When comparing various mixes, B3 and B3-LWA demonstrate similar behavior. However, the incorporation of silica sand in B3-SS significantly increases thermal conductivity, rendering it the least desirable option as a thermal insulating material. These observations align with the findings of Kim et al. [52], which indicated that the addition of aggregate in cement paste raises thermal conductivity and an increased volume fraction of normal-weight aggregate yields higher thermal conductivity for concrete. Among coarse and fine aggregates, the influence of fine aggregate is more pronounced.

**Figure 6:**
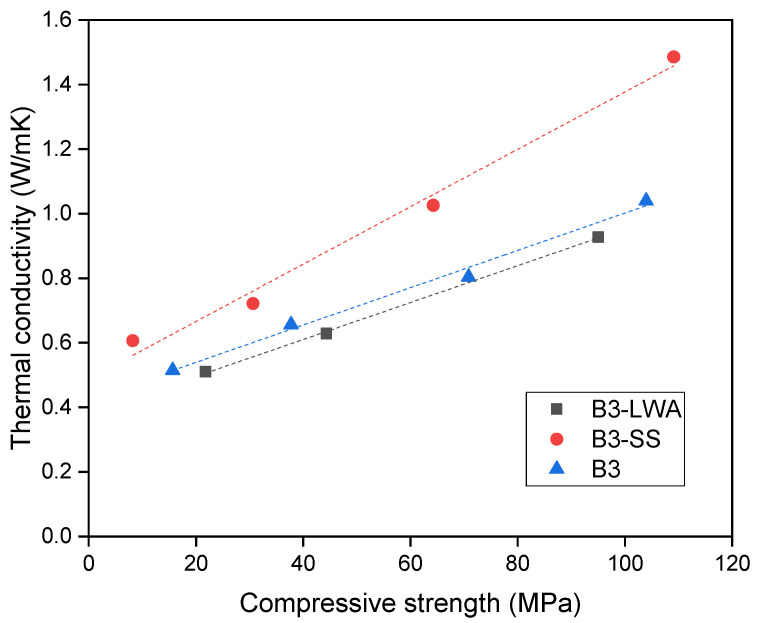
Thermal conductivity vs. compressive strength plot.

The thermal conductivity is then plotted against the compressive strength of concrete to differentiate the behavior between B3 and B3-LWA (Figure 6). In general, B3-LWA outperforms B3, as it exhibits lower thermal conductivity at each strength level within the investigated range. Consequently, B3-LWA proves to be more efficient in thermal insulation, which can be attributed to the differences in internal pore structure, as discussed in Section 3.1. This observation proves that B3-LWA is a more appealing choice for applications requiring both structural and thermal performance.

### 3.3. Drying Shrinkage

**Figure 7:**
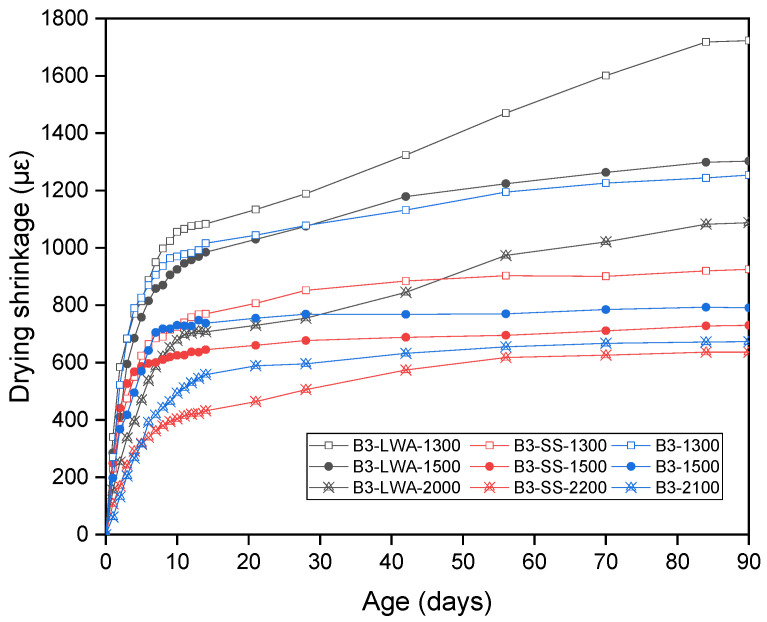
Drying shrinkage measurements up to 90 days.

This section presents the 90-day measurement of drying shrinkage for 9 groups of test specimens made with different mix compositions (before foam addition) and foam contents (Figure 7). As shrinkage development is consistent among specimens within the same group throughout the measuring period, the average results are presented. The first observation from Figure 7 is that the drying shrinkage decreases as density increases, indicating that a higher foam volume increases drying shrinkage. Moreover, the effect appears to be more pronounced at lower densities. There is a sharp decrease in shrinkage when density increases from 1300 kg/m^3^ to 1500 kg/m^3^. Between 1500 kg/m^3^ and 2000 kg/m^3^, the decrease in shrinkage becomes more gradual. The second point to note is the effect of mixed composition. In line with existing research, using silica sand helps to suppress the development of shrinkage compared to pure cement binders. However, the addition of lightweight aggregate has been shown to significantly increase drying shrinkage. Due to high porosity and low stiffness, the lightweight aggregate itself is likely to undergo deformation during the drying process, leading to higher shrinkage values. Unlike B3 and B3-SS, where shrinkage stabilizes after 50–60 days, the shrinkage of B3-LWA continues to develop until the age of 84 days. These observations suggest that lightweight concrete, particularly with lightweight aggregates, is more prone to higher shrinkage. However, to fully understand the implications of this shrinkage, it is necessary to consider the potential for shrinkage cracking. The following section addresses this crucial aspect, providing a more comprehensive evaluation of the material’s behavior under drying shrinkage.

### 3.4. Potential for Shrinkage Cracking Evaluation

To assess the actual risk of cracking induced by drying shrinkage, it is necessary to estimate the shrinkage cracking potential of the material. This involves calculating the stress induced by shrinkage (assuming full restraint) and comparing it to the tensile strength of the concrete (or flexural stress, as only qualitative comparison among different mixes is necessary).

Table 3 presents the flexural stress ($\sigma_{f}$) of each concrete batch, obtained in Section 3.1, along with the modulus of elasticity (*E*) and 28-day shrinkage ($\varepsilon_{s}$) of the samples. Assuming that concrete materials behave elastically before the occurrence of cracks, the shrinkage stress ($\sigma_{s}$) can be calculated using the elastic modulus (E) and shrinkage strain (ε) as follows:

(1) $$\sigma_{s}=E\varepsilon_{s}$$

The potential of shrinkage cracking ($P_{c}$) is defined as the ratio of shrinkage stress ($\sigma_{s}$) to flexural strength ($\sigma_{f}$):

(2) $$P_{c}=\sigma_{s}{/\sigma }_{f}$$

While $P_{c}$ cannot directly represent the probability of cracking, it serves as an indicator to compare the shrinkage cracking potentials among various mixes. A higher $P_{c}$ value implies an increased potential for shrinkage crack formation due to a larger disparity between the shrinkage stress and flexural strength. According to the existing literature and Code of Practice, the flexural strength and elastic modulus for concrete with different compressive strengths can be calculated as follows [53,54]:

(3) $$\sigma_{f}=0.45\cdot \sigma_{c}^{2/3}$$

(4) $$E=3.46\sqrt{\sigma_{c}}+3.21$$

**Figure 8:**
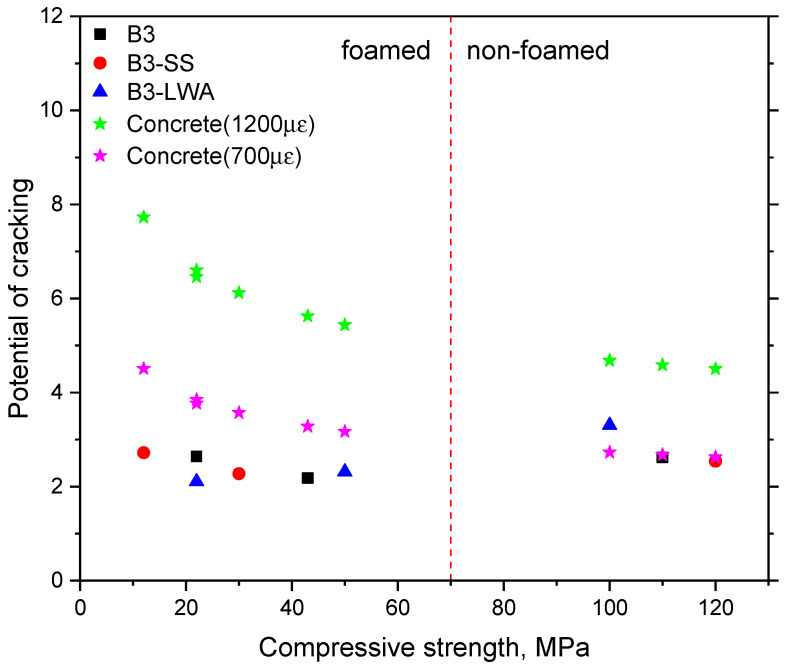
The potential of shrinkage cracking evaluation ($P_{s}$) as the shrinkage stress/flexural stress ratio.

The shrinkage of concrete/mortar materials is influenced by various factors such as water/cement ratio, type of aggregate, and ambient humidity [55]. According to data from the Northwestern University Concrete Shrinkage Database, the 28-day shrinkage for concrete/mortar with a water/cement ratio of 0.35 generally falls within 700–1200 με [56,57]. Considering normal concrete/mortar with similar compressive strength to the foamed concrete used in this experiment, we calculated the $P_{c}$ values for normal concrete at empirical shrinkage values (700–1200 με). These results are presented in Figure 8.

For normal concrete, lower shrinkage at the same compressive strength and Young’s modulus correlate with reduced cracking potential. Hence, we compared the computed cracking potential ($P_{c}$) of the specimens in this study with that of normal concrete exhibiting shrinkage of 700 με. In the absence of foam, the $P_{c}$ of the B3-LWA group with lightweight aggregate was 121% higher than that of normal concrete (700 με). The $P_{c}$ of the B3 and B3-SS groups differed by less than 10% compared to normal concrete (700 με). With the addition of foam, the $P_{c}$ of all foamed mixes was approximately 55–73% of normal concrete (700 με), indicating a lower shrinkage cracking potential in foamed concrete. This can be attributed to foamed concrete having a lower elastic modulus, which reduces shrinkage stress under comparable compressive strength and shrinkage levels. Thus, the judicious use of foam can effectively mitigate the potential of shrinkage cracking in concrete at the same compressive strength level. This underscores the importance of considering multiple factors (shrinkage, strength, and elasticity) when assessing cracking risk, rather than focusing solely on shrinkage values.

Despite the lower cracking potential in foamed mixes, minimizing shrinkage remains beneficial for overall concrete performance and durability. Future research could explore the incorporation of shrinkage-reducing admixtures (SRAs) or the replacement of Ordinary Portland cement with calcium sulfoaluminate (CSA) cement to further optimize mix designs and reduce drying shrinkage.

## 4. Cooling and Heating Load Assessment of a Typical Modular Building Using EnergyPlus Simulation

The proposed use of lightweight concrete can contribute to the reduction of building energy consumption and the associated environmental impact. This is mainly achieved by reducing the cooling and heating loads required to maintain a comfortable indoor temperature. By incorporating foamed concrete in both the roof and external walls, heat transfer between the interior and exterior of the building envelope can be reduced, resulting in a decreased demand for HVAC (heating, ventilation and air conditioning) systems and improved energy efficiency.

To assess the impact of the proposed lightweight concrete on building energy consumption during the operational phase, the annual cooling and heating load of a typical multi-storey modular building was analyzed using the building energy simulation program EnergyPlus, with input material properties measured from the previous sections. Developed by the US Department of Energy, EnergyPlus is a powerful whole-building energy simulation tool used by researchers worldwide for modeling and simulating a building’s energy flow including heating, cooling, lighting, and ventilation. The software has been widely tested and validated by local researchers in Hong Kong [58,59,60,61,62,63,64,65,66].

**Figure 9:**
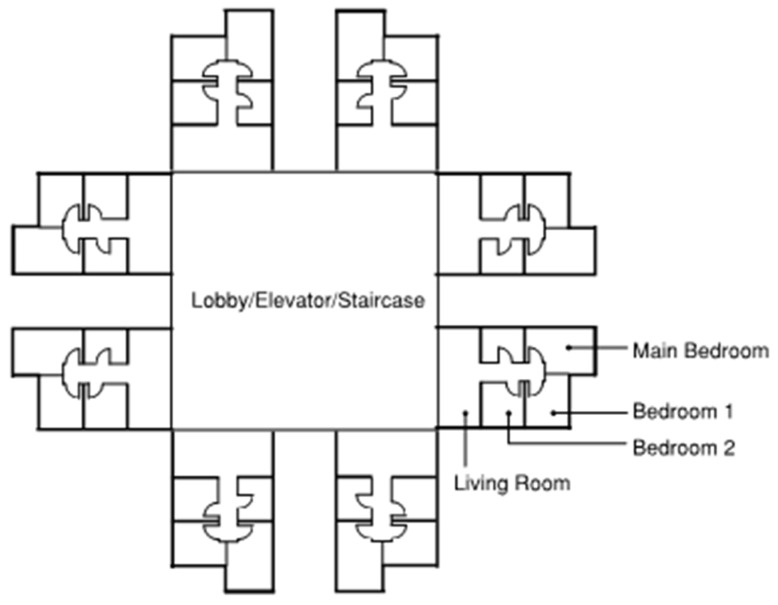
Schematic layout of a typical floor in MiC residential building.

**Figure 10:**
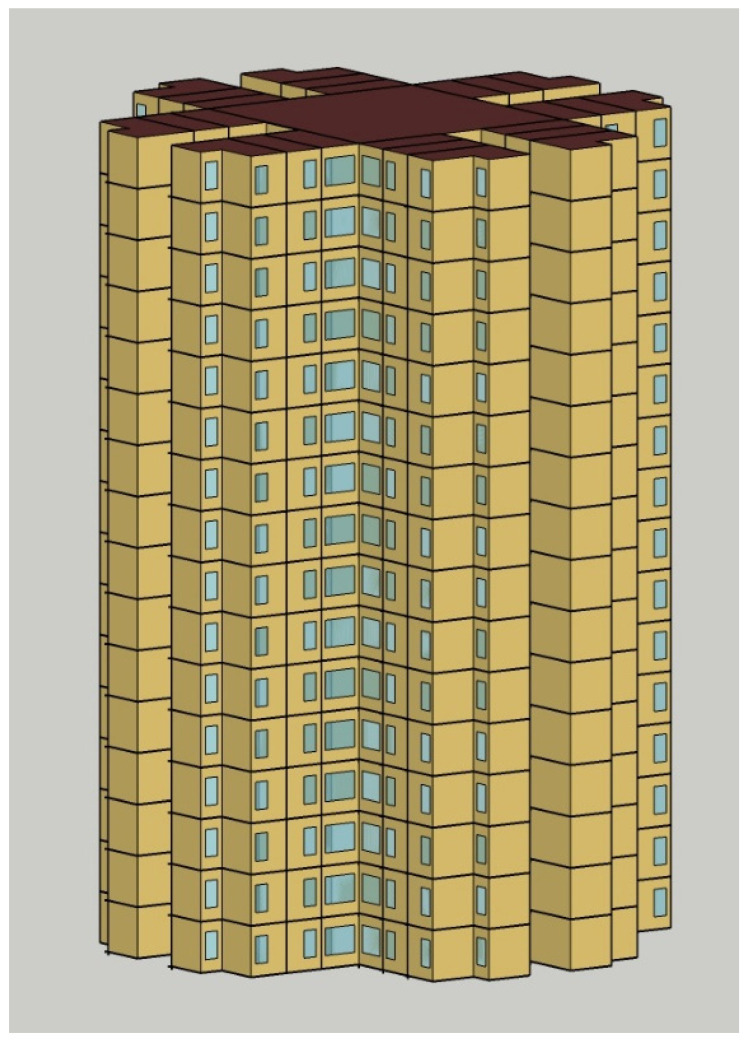
3D model of a MiC residential building, simulated in EnergyPlus.

A 16-storey residential modular building with a generic layout design (Figure 9) was adopted for building energy simulation. Figure 10 shows the model used for the simulations, which was created in SketchUp drawing software and integrated into the EnergyPlus engine using the OpenStudio plug-in. There are eight identical apartments per floor, with an even distribution among the North, East, South, and West-facing sides. Each apartment contains one living room and three bedrooms, which are air-conditioned zones. To simulate the cooling and heating loads, the internal heat gained from various sources within the building, including people, lighting, and electric equipment, must be correctly included in the model. With reference to the works of local researchers [66,67,68,69,70,71], the occupancy density, lighting and equipment power intensities, and the corresponding operating schedule adopted in this study are listed in Table 4 and Table 5.

The configuration of the external wall and window glazing was based on previous research on local public rental housing [64,65,66,68,72], as detailed in Table 6 and Table 7. The external walls have a composite structure with several layers. The benefits of replacing conventional concrete with foamed concrete of varying density levels (corresponding to thermal conductivities of 0.5, 0.8, 1.1, and 1.4 W/mK) in the building envelope system of the multi-storey residential building were then assessed by evaluating its impact on annual cooling and heating loads. To obtain a more comprehensive understanding of the trend, the simulation also included hypothetical lightweight concrete with thermal conductivities of 1.7 and 2.0 W/mK. In principle, the characteristics of a building’s roof are also relevant factors. However, in contrast to single-storey buildings, the impact of roof material on multi-storey buildings like this one is relatively minor. As a result, the default roof material specified in EnergyPlus was utilized in the analysis.

**Figure 11:**
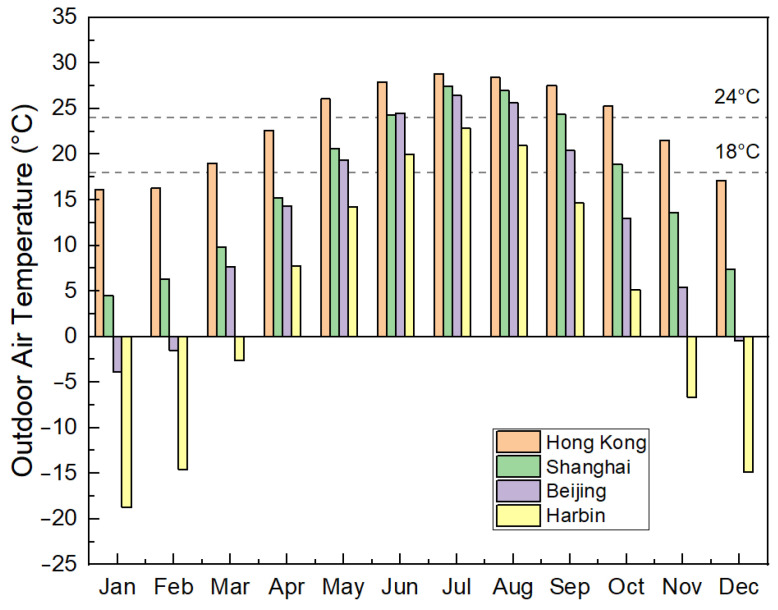
Monthly outdoor air temperature trends in Hong Kong, Shanghai, Beijing, and Harbin.

While our main focus lies in the application of lightweight concrete for residential buildings in Hong Kong, examining the potential influence of building envelopes with different concrete thermal conductivity across various climate zones can be insightful. To this end, the developed building model was subjected to simulations using weather data from three additional Chinese cities: Harbin, Shanghai, and Beijing. Figure 11 displays the monthly outdoor temperatures for the cities under investigation.

### 4.1. Hong Kong

Located in the subtropical region of the northern hemisphere (22.3 °N, 114.17 °E), Hong Kong has a long, hot, and humid summer and short and mild winter. There is a substantial demand for air conditioning for residential flats, especially over the summer cooling months (April to October) [69,72]. Even during the winter season, Hong Kong may experience occasional high outdoor temperatures that require cooling measures [73]. Due to the high building density in Hong Kong, residents often rely on air conditioning despite some building blocks being designed to optimize indoor thermal conditions with natural ventilation and prevailing winds. This is primarily due to the negative impact of traffic-induced noise pollution and poor air quality on the indoor environment [64]. On the other hand, the use of heating systems is quite rare [64]. The dominance of cooling demand overheating is also evidenced by the calculated degree-day (a measure of how much cooling/heating is needed to maintain a comfortable indoor temperature) of 1405 for cooling and 162 for heating, using 26 °C and 18 °C as the base temperatures [74].

The simulation utilized a Typical Meteorological Year (TMY) hourly weather file for Hong Kong [75] to account for the long-term typical weather conditions, including temperature, humidity, solar radiation, and wind. As the specific HVAC system for the building was not known, a simplified Ideal Loads Air System model was adopted to estimate the cooling loads based on the thermal characteristics of the building envelopes made with different materials. The model assumed a design indoor temperature of 24 °C. While more complex HVAC systems, such as VAV and VRF, can consider various factors including duct leakage, equipment sizing, and control strategy, the Ideal Loads Air System provides an efficient and straightforward method for cooling load calculation.

**Figure 12:**
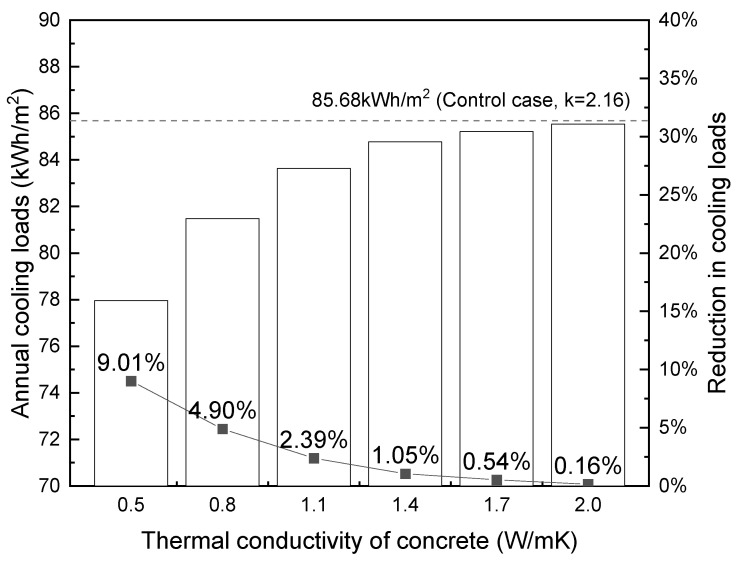
Influence of thermal conductivity on building cooling loads (per m^2^) in Hong Kong.

Based on the above inputs, Figure 12 shows the annual cooling loads per meter square of the building calculated by EnergyPlus (left y-axis). The y-axis on the right shows the percentage of reduction of annual cooling loads compared to the base case with k = 2.16. As expected, the cooling loads increase with thermal conductivity. The highest reduction in cooling load consumption was observed with foamed concrete with a thermal conductivity of 0.5, which resulted in a 9.01% reduction compared to the control case. The reduction in cooling loads decreased as the thermal conductivity of the foamed concrete increased, with a reduction percentage of 0.16% occurring when thermal conductivity equals to 2.0. It is also noted that the savings of cooling loads are not linearly increasing with decreased thermal conductivity within the investigated range. The beneficial effect is more pronounced at lower conductivity levels, similar to observations in Ref. [76].

### 4.2. Harbin

**Figure 13:**
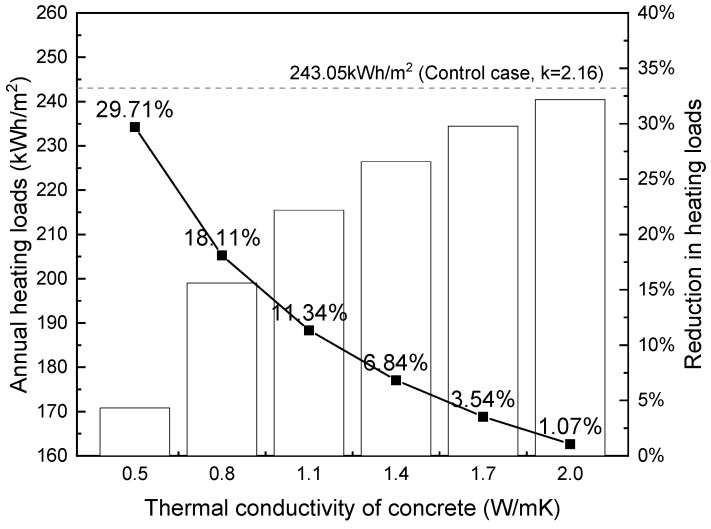
Influence of thermal conductivity on building heating loads (per m^2^) in Harbin.

In this section, the same building model was simulated using the Harbin weather file. Harbin (45.75 °N, 126.77 °E), located in the northeastern part of China, experiences well-defined seasons including a long, cold winter and a short, cool summer. During winter months, the city experiences severe cold, with average temperatures well below freezing and occasional extreme lows of −35 °C or lower, necessitating extensive heating to maintain comfortable indoor temperatures. On the other hand, the demand for cooling is quite low. To estimate the annual heating loads, the indoor design temperature was set to 18 °C, according to the acceptable indoor temperature proposed by Ref. [77]. The calculated annual heating loads against the thermal conductivity of foamed concrete are plotted in Figure 13.

Figure 13 shows that reducing the thermal conductivity of concrete that composed of the building’s external wall leads to considerable savings in annual heating loads in multi-storey residential blocks in Harbin. Using foamed concrete with 0.5 W/mK thermal conductivity, about a 30% reduction in annual heating loads can be achieved. Similar to Figure 12, a non-linear relationship exists between conductivity and heating loads.

### 4.3. Shanghai and Beijing

**Figure 14:**
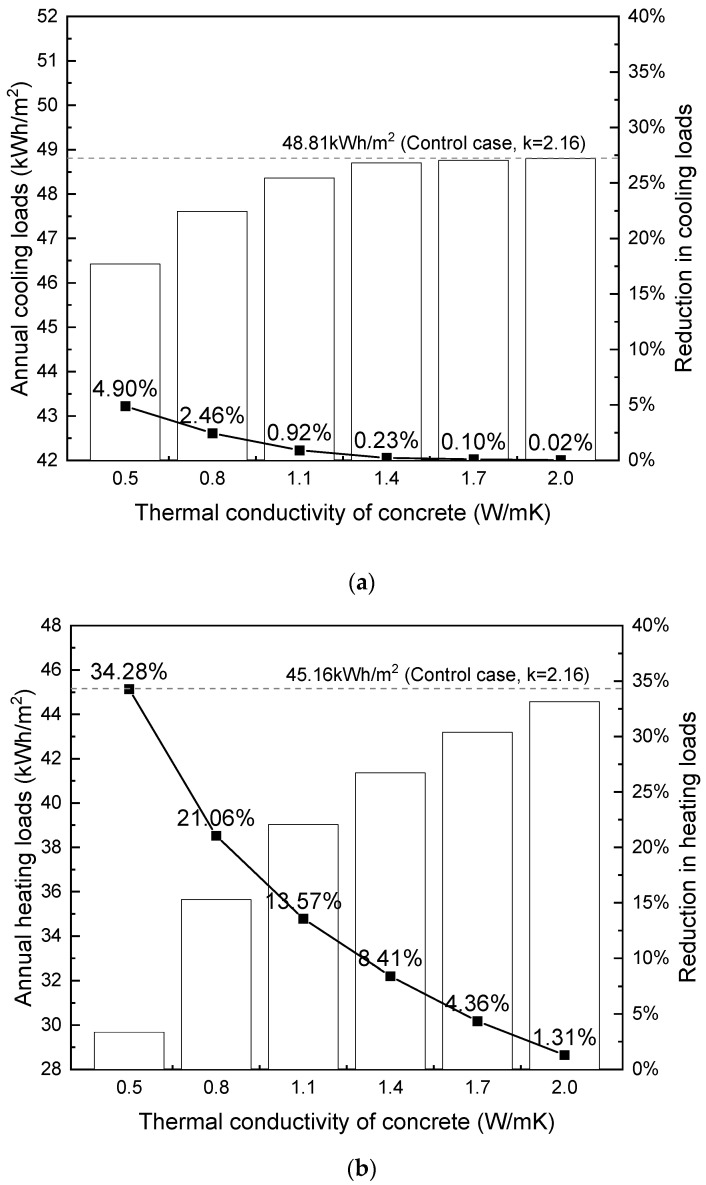
Influence of thermal conductivity on building loads (per m^2^) in Shanghai: (**a**) cooling; (**b**) heating.

**Figure 15:**
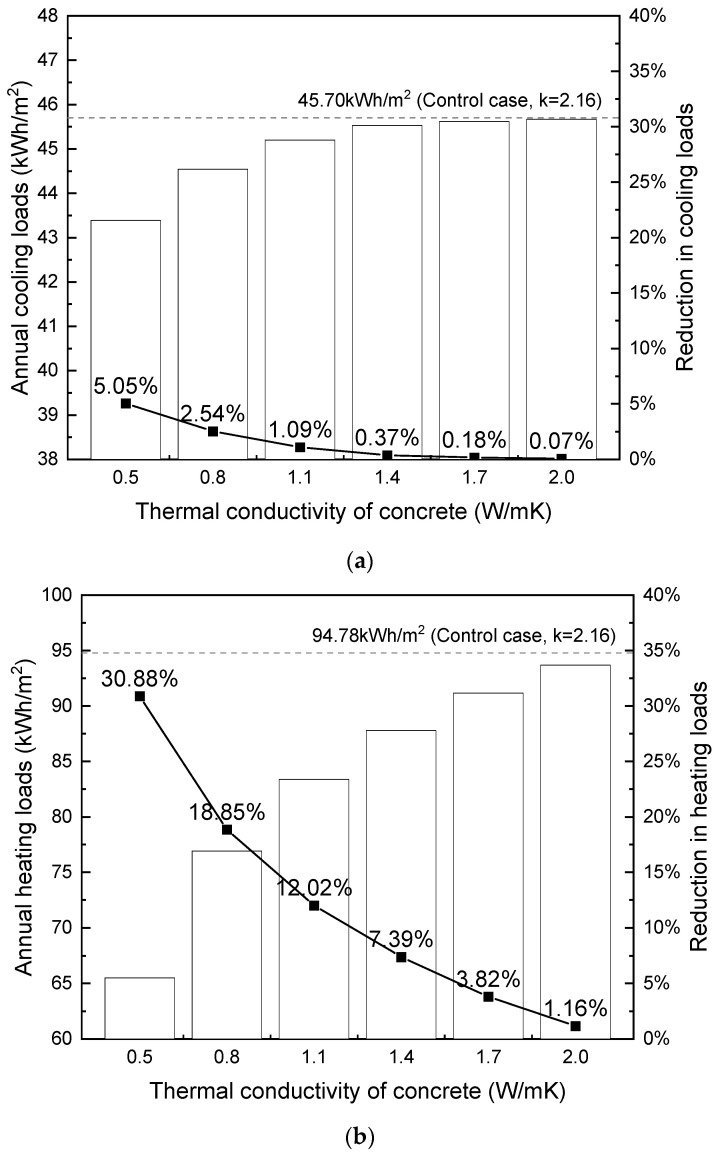
Influence of thermal conductivity on building loads (per m^2^) in Beijing: (**a**) cooling; (**b**) heating.

According to the Code for Thermal Design of Civil Buildings (GB 50176-2016) [78], Shanghai (31.4 °N, 121.45 °E) and Beijing (39.8 °N, 116.47 °E) are classified into ‘hot summer and cold winter region’ and ‘cold region’, respectively. Shanghai’s humid subtropical climate features distinct seasons, with warm, wet springs, hot, humid summers, mild autumns, and chilly, damp winters. On the other hand, Beijing’s temperate continental climate exhibits hot, humid summers and cold, dry winters. Both cities require cooling during the hot summer months and heating during the cold winter months to maintain thermal comfort. For the simulation, the thermal comfort temperature range was set at 18 °C–24 °C. Figure 14 and Figure 15 depict the annual cooling and heating loads in relation to the thermal conductivity of foamed concrete. 

In accordance with the findings presented in Section 4.1 and Section 4.2, the results in Figure 14 and Figure 15 also demonstrate that decreasing concrete thermal conductivity leads to savings in both cooling and heating loads for the building. This relationship remains non-linear, with a more pronounced impact observed at lower conductivity levels. The sensitivity of heat flow to changes in conductivity is greater at lower levels, particularly in the absence of supplementary insulation layers within the external wall composite, resulting in a more pronounced effect of varying conductivity. Conversely, altering conductivity at higher levels has a negligible effect on total heat flow because high thermal conductivity leads to an insignificant temperature gradient across the building envelope. Consequently, even if conductivity is further increased, the cooling/heating loads remain relatively unchanged due to the plateauing of heat flow.

From Figure 12 through Figure 15, it is noted that the thermal conductivity of the building envelope has a considerably greater impact on annual heating loads than cooling loads in terms of percentage of reduction, This finding is consistent with the existing literature [32]. During the winter months, the external walls of buildings are exposed to the cold outdoor temperatures, which can result in significant heat loss through the walls from the inside to the outside. The use of building materials with lower thermal conductivity can reduce this heat loss and make it easier to maintain a comfortable indoor temperature with less energy consumption. On the other hand, during the summer months, heat gain through the walls is less of an issue, as the cooling load is primarily driven by solar gain through windows and internal heat sources. Therefore, the wall material has a relatively smaller impact on cooling energy demand compared to heating energy demand.

Based on the results, it is concluded that using the proposed lightweight concrete B3-LWA with a density between 1300–1500 kg/m^3^ (corresponding to a thermal conductivity between 0.5 to 0.6 W/mK) with compressive strength between 20 MPa to 45 MPa can significantly reduce energy usage in buildings. Specifically, it can decrease cooling loads by 7% to 9% in multi-storey residential buildings in Hong Kong. It can potentially generate even greater energy savings on heating loads for buildings in cold climates. In particular, the use of B3-LWA can enable up to 30% reductions in heating loads for similar buildings situated in areas with cold winters such as Shanghai, Beijing, and Harbin, highlighting its potentially higher impact and value for cold region applications.

It should be noted that the calculated cooling/heating loads derived from the use of the Ideal Loads Air System in this study do not necessarily equate to the actual electricity consumption of the HVAC system. This is because the Ideal Loads Air System operates at 100% efficiency with a COP equal to 1, which is unrealistic. However, it is sufficient for making relative comparisons between different building envelope systems. This investigation, which utilized a relatively simple structure with several assumptions and simplifications, highlighted the potential thermal performance benefits of using the proposed lightweight concrete in the building envelope of a residential block in different climate zones. To obtain more reliable and accurate results on building energy consumption, it would be necessary to refine the input data provided to the simulation, which is beyond the scope of this study.

## 5. Conclusions

In this study, experimental tests on mechanical strength, drying shrinkage, and thermal conductivity of lightweight concrete were conducted. The effects of aggregate type and foam content were investigated. The potential impact of adopting the proposed lightweight concrete on the energy efficiency of a multi-storey residential modular building was also explored using EnergyPlus simulation. The following conclusions are highlighted:

1. The compressive strength and the flexural strength of the concrete mixes were found to be almost linearly related to density within the studied range. B3-LWA (with the combined use of lightweight aggregate and foam) demonstrated the highest strength-to-density ratio, making it the best-performing mix among the three.
2. At a specific strength level, B3-LWA exhibited lower thermal conductivity compared to B3 (made with foam only), while B3-SS had significantly higher thermal conductivity due to the incorporation of silica sand into foamed concrete. This finding is consistent with previous research, which showed that normal-weight fine aggregate has a pronounced effect on thermal conductivity.
3. The drying shrinkage decreased as concrete density increased. The effect of varying foam volume content was more pronounced at lower density ranges (between 1300 kg/m^3^ and 1500 kg/m^3^). The addition of lightweight aggregate significantly increased drying shrinkage. On the contrary, incorporating silica sand provided a good restraining effect, and B3-SS maintained much lower shrinkage levels even with a high volume of foam.
4. Despite higher drying shrinkage in some lightweight mixes, incorporating foam in concrete can effectively reduce the risk of shrinkage-induced cracking. Analysis of the cracking potential revealed that foamed mixes exhibited 55–73% of the cracking potential of normal concrete. This reduction is primarily attributed to the lower Young’s modulus of foamed concrete, which allows the material to better accommodate shrinkage strains. Consequently, the strategic use of foam can enhance concrete’s resistance to shrinkage-induced cracking at the same compressive strength level.
5. The results of building energy simulations suggested that using the proposed lightweight concrete with low thermal conductivity in external walls can be an effective means to reduce building energy consumption related to cooling and heating. Between the two, the impact on heating loads is more pronounced.

The strength test results demonstrate that the proposed lightweight concrete, particularly B3-LWA with densities between 1300 and 1500 kg/m^3^, has great potential in various structural applications. Achieving strengths between 20 MPa to 45 MPa, it is suitable for nominal structural use, especially in modular construction features such as double walls and double slabs. Its excellent thermal insulation properties further enhance its appeal, offering significant reductions in energy consumption and greenhouse gas emissions by minimizing cooling and heating requirements. This combination of structural integrity and thermal efficiency makes it valuable beyond modular construction. The lightweight concrete is well-suited for projects with weight constraints, like renovations of existing structures or tall buildings, and for manufacturing prefabricated elements, aligning with trends in off-site construction. Its enhanced thermal properties make it an excellent choice for external walls in both residential and commercial buildings, contributing to overall energy efficiency. By addressing multiple challenges in modern construction—from structural requirements to energy conservation and sustainable practices—this lightweight concrete offers a comprehensive solution for the evolving needs of the construction industry.

## Figures and Tables

**Table 1:** Chemical compositions of OPC, silica fume, fly ash and lightweight aggregate.

	OPC ^1^	SF ^2^	FA ^3^	LWA ^4^
CaO	68.9%	0.6%	6.5%	28.3%
SiO_2_	18.0%	98.9%	55.3%	40.0%
Al_2_O_3_	4.0%	-	25.7%	22.7%
Fe_2_O_3_	2.9%	0.1%	6.5%	4.2%
SO_4_	4.6%	-	1.1%	1.9%
K_2_O	0.8%	0.4%	3.1%	0.8%
TiO_2_	0.4%	-	1.6%	1.3%
MnO	0.1%	-	0.2%	0.1%
MgO	-	-	-	0.7%

^1^ Ordinary Portland cement. ^2^ Silica fume. ^3^ Fly ash. ^4^ Lightweight aggregate.

**Table 2:** Mix proportions of lightweight concrete (in kg/m^3^) before foam addition.

	Cement	Silica Fume	Fly Ash	Aggregate	Water
B3-LWA	763	85	424	424 (lightweight aggregate)	297
B3-SS	843	94	468	468 (silica sand)	328
B3	1021	113	567	0	397

**Table 3:** Parameters relevant to the potential for shrinkage crack formation.

Items	Density: 1300 kg/m^3^			Density: 1500 kg/m^3^			Density: 2000–2200 kg/m^3^		
	B3	B3-SS	B3-LWA	B3	B3-SS	B3-LWA	B3	B3-SS	B3-LWA
Shrinkage ($\varepsilon_{s}$)/με	1078	852	1189	769	677	1075	596	506	755
Flexural strength ($\sigma_{f}$)/MPa	2.1	1.3	2.9	4.1	3.1	5.6	8.6	9.1	6.9
Modulus (*E*)/GPa	5.1	4.2	5.1	11.5	10.6	12.1	37.8	45.7	30.2
Shrinkage stress ($\sigma_{s}$)/MPa	5.5	3.5	6.1	8.9	7.2	13.0	22.5	23.1	22.8

**Table 4:** Adopted parameters in building energy simulation.

Parameters	Value
Floor height	2.8 m
Total floor area per floor	515 m^2^
Conditioned floor area per floor	326 m^2^
Occupancy density	12 m^2^/person or 0.083 person/m^2^
Occupancy load	100 W/person
Lighting load	15 W/m^2^ (living room); 13 W/m^2^ (bedroom)
Equipment load	142 W/room (living room); 45 W/room (bedroom)

**Table 5:** Schedule of building operation.

Hour	Occupancy		Lighting	Equipment	Cooling	
	Living Room	Bedroom			Living Room	Bedroom
1	0	1	0	0.2	0	1
2	0	1	0	0.2	0	1
3	0	1	0	0.2	0	1
4	0	1	0	0.2	0	1
5	0	1	0	0.2	0	1
6	0	1	0	0.2	0	1
7	0	1	0.3	0.37	0	1
8	0	0	0.5	0.54	0	0
9	0	0	0.3	0.54	0	0
10	0.33	0	0	0.54	1	0
11	0.33	0	0	0.54	1	0
12	0.33	0	0	0.54	1	0
13	0.33	0	0	0.54	1	0
14	0.33	0	0.5	0.63	1	0
15	0.33	0	0	0.43	1	0
16	0.33	0	0	0.43	1	0
17	0.33	0	0	0.43	1	0
18	0.33	0	0	0.43	1	0
19	0.33	0	0.5	0.43	1	0
20	1	0	1	1	1	0
21	1	0	1	1	1	0
22	1	0	1	1	1	0
23	0	1	1	1	0	1
24	0	1	0.5	1	0	1

**Table 6:** Thermo-physical properties of external walls.

	Thermal Conductivity (W/mK)	Density (kg/m^3^)	Thickness (mm)
Mosaic tile (outside layer)	1.5	2500	5
Sand Plaster	0.72	1860	10
Heavy concrete	2.16	2400	100
Gypsum plaster (inside layer)	0.38	1120	10
Mosaic tile (outside layer)	1.5	2500	5
Sand Plaster	0.72	1860	10
Foamed concrete	0.5–2.0	1300–2200	100
Gypsum plaster (inside layer)	0.38	1120	10

**Table 7:** Thermo-physical properties of window glazing material.

	U-Value (W/m^2^K)	Solar Heat Gain Coefficient
Fenestration/window	5.69	0.57

## Data Availability

Data will be provided upon request.
